# Deciphering the Enhancing Impact of Exogenous Brassinolide on Physiological Indices of Melon Plants under Downy Mildew-Induced Stress

**DOI:** 10.3390/plants13060779

**Published:** 2024-03-09

**Authors:** Tai Liu, Huichun Xu, Sikandar Amanullah, Zhiqiang Du, Xixi Hu, Ye Che, Ling Zhang, Zeyu Jiang, Lei Zhu, Di Wang

**Affiliations:** 1 Daqing Branch of Heilongjiang Academy of Agricultural Sciences, Daqing 163711, China; liutai1997@sina.com (T.L.); xhc836060@sina.com (H.X.); duzhiqiang2023@sina.com (Z.D.); huxixi116@sina.com (X.H.); cheye12345@sina.com (Y.C.); zhang8374ling@sina.com (L.Z.); 18845922229@163.com (Z.J.); 15246088325@163.com (L.Z.); 2 College of Horticulture and Landscape Architecture, Northeast Agricultural University, Harbin 150030, China; 3 Key Laboratory of Biology and Genetic Improvement of Horticulture Crops (Northeast Region), Ministry of Agriculture and Rural Affairs, Northeast Agricultural University, Harbin 150030, China

**Keywords:** brassinolide, downy mildew, antioxidant enzymes, photosynthesis, *Cucumis melo* L., *Pseudoperonospora cubensis*

## Abstract

Melon (*Cucumis melo* L.) is a valuable horticultural crop of the Cucurbitaceae family. Downy mildew (DM), caused by *Pseudoperonospora cubensis*, is a significant inhibitor of the production and quality of melon. Brassinolide (BR) is a new type of phytohormone widely used in cultivation for its broad spectrum of resistance- and defense-mechanism-improving activity. In this study, we applied various exogenous treatments (0.5, 1.0, and 2.0 mg·L^−1^) of BR at four distinct time periods (6 h, 12 h, 24 h, and 48 h) and explored the impact of BR on physiological indices and the genetic regulation of melon seedling leaves infected by downy-mildew-induced stress. It was mainly observed that a 2.0 mg·L^−1^ BR concentration effectively promoted the enhanced photosynthetic activity of seedling leaves, and quantitative real-time polymerase chain reaction (qRT-PCR) analysis similarly exhibited an upregulated expression of the predicted regulatory genes of photosystem II (PSII) *CmHCF136* (*MELO3C023596.2*) and *CmPsbY* (*MELO3C010708.2*), thus indicating the stability of the PSII reaction center. Furthermore, 2.0 mg·L^−1^ BR resulted in more photosynthetic pigments (nearly three times more than the chlorophyll contents (264.52%)) as compared to the control and other treatment groups and similarly upregulated the expression trend of the predicted key enzyme genes *CmLHCP* (*MELO3C004214.2*) and *CmCHLP* (*MELO3C017176.2*) involved in chlorophyll biosynthesis. Meanwhile, the maximum contents of soluble sugars and starch (186.95% and 164.28%) were also maintained, which were similarly triggered by the upregulated expression of the predicted genes *CmGlgC* (*MELO3C006552.2*), *CmSPS* (*MELO3C020357.2*), and *CmPEPC* (*MELO3C018724.2*), thereby maintaining osmotic adjustment and efficiency in eliminating reactive oxygen species. Overall, the exogenous 2.0 mg·L^−1^ BR exhibited maintained antioxidant activities, plastid membranal stability, and malondialdehyde (MDA) content. The chlorophyll fluorescence parameter values of F0 (42.23%) and Fv/Fm (36.67%) were also noticed to be higher; however, nearly three times higher levels of NPQ (375.86%) and Y (NPQ) (287.10%) were observed at 48 h of treatment as compared to all other group treatments. Increased Rubisco activity was also observed (62.89%), which suggested a significant role for elevated carbon fixation and assimilation and the upregulated expression of regulatory genes linked with Rubisco activity and the PSII reaction process. In short, we deduced that the 2.0 mg·L^−1^ BR application has an enhancing effect on the genetic modulation of physiological indices of melon plants against downy mildew disease stress.

## 1. Introduction

Melon (*Cucumis melo* L.) is known as a popular fruit crop due to its high demand role in the commercial fruit crop industry. However, some pests and diseases negatively affect the yield and quality of melon. Downy mildew (DM), caused by an obligate biotrophic oomycete (*Pseudoperonospora cubensis*) is one of the main foliar diseases in cucurbit crops, affecting over 40 cucurbit species, including melon [1]. Thomas et al. [2] reported that the genetic variability of *P. cubensis* is classified into 10 pathotypes by their pathogenicity in numerous cucurbit species, most of which have been identified as being infectious to melon (*C. melo* L., var. *reticulatus*).

In China, the high hydrothermal conditions in the summer are beneficial for the dispersion and germination of DM spores in melon plants. Encysted spores can survive in the topsoil and are ready to spread in the next production season [3]. In cucumber, DM disease usually causes a reduction in the total production of yield and plant biomass [4,5]. The manifestation of downy mildew has negatively impacted cucumber production in over 80 nations and areas worldwide [6]. Thus far, there have not been any documented cases of widespread DM outbreaks on melon plants where the fungus severely damaged plants and reduced fruit quality and production [7].

In plant–pathogen interactions, DM-susceptible plants show chlorotic and angular patches on upper leaf surfaces. As the disease progresses, the chlorotic spots become bigger, turn necrotic, and eventually cause leaf mortality [3]. The chlorosis of leaves mainly leads to a downregulated photosynthetic rate, decreased chlorophyll biosynthesis, a blockage of carbon dioxide (CO_2_) fixation and carbohydrate metabolism pathways, and ultimately contributes to the effective growth of spores due to a lack of nutrients [8,9]. Similarly, pathogen infestation induces the downregulation of Rubisco-associated regulatory genes, as well as the super-oxidized membrane of the cuticle’s photosynthetic apparatus, which severely causes a decrease in assimilatory efficiency in leaves [10].

The plant cell membrane acts as a defensive barrier against biotic and abiotic factors [11]. There are three types of plant-membrane-related lipids that have been categorized based on their specific components and configuration, and glycerolipids are known as primary lipids composed of differentiated fatty acids in the structure of glycerol [12,13,14,15]. Digalactosyldiacylglycerol (DGDG) and monogalactosediacylglycerol (MGDG) are known as major lipids located in the green tissues of plants (leaves) and help to regulate the chloroplast structure and chlorophyll pigment synthesis [16]. Membrane lipids become altered through the basic phenomenon of remodeling due to various environmental fluctuations [17]. The DGDG level increases during stress conditions, and the stability of the cell membrane becomes disturbed due to the irregular proportion of MGDG to DGDG [18,19,20,21].

Physiological and molecular regulations act as the major modulatory pathways against stress in crop plants [22]. In general, physiological regulation alters the morphological attributes of plants by triggering hormonal distribution to alleviate stress-induced damage, e.g., the endogenous activity of auxin promotes the development of root architecture, while stress conditions effectively alter the internal biosynthesis in roots [23]. Meanwhile, maintained peroxidase (POD) and superoxide dismutase (SOD) activities normalize the extreme reactive oxygen species (ROS) through internal enzymatic functions and relieve the super-oxidized structure of membranes affected by stress [24]. Thus, regulated relative conductivity (REC), malondialdehyde (MDA) content, and active antioxidants are helpful for improving the level of cell damage caused by stress [25]. Nevertheless, studies have been more focused on understanding complex molecular mechanisms at the translational and transcriptional levels than on physiological regulation, and these have pinpointed functional candidate genes, encoding proteins, and modulated pathways regulating hormonal biosynthesis and the remodeling of cell membrane activity against plant stress tolerance [26,27,28].

The significant adaptation of the melon plant to stress conditions mainly relies on regulatory genes contributing photosynthetic pigment content, carbohydrates (osmotica), and antioxidant activities. Some previous studies reported that the *Glgc* gene (encoding AGPase, the key enzyme in starch biosynthesis) and the *SPS* (sucrose phosphate synthase) gene act as rate-limiting enzyme genes for the biosynthesis of starch and sucrose and play an essential role in energy synthesis during growth and development in plants, as well as improving fruit quality [29,30]. Similarly, upregulation of the *PEPC* (phosphoenolpyruvate carboxylase) gene in transgenic rice showed an increased fraction of photosynthetically fixed carbon allocated to the organic acid to resist nutrient deficiency [31]. PSII in plants always suffers from photoinhibition during stress conditions [32,33], while *HCF* (high chlorophyll fluorescence) and the *Psb* gene effectively trigger the stabilization of PSII and promote the regular formation of chloroplasts and thylakoids [34,35]. In addition, the *LHCP* gene (light harvesting chlorophyll a/b binding protein), located in PSII, is an integral component of the antenna chlorophyll complex, and the *CHLP* gene, which could catalyze the precursor substance for chlorophyll biosynthesis, jointly controls the reaction process of chlorophyll [36,37].

At present, the main strategy to control downy mildew disease relies on synthetic pesticides, but these pesticides contain harmful chemicals that affect the environment and fruit quality and are also detrimental to human health [38]. Thus, it highlights the necessity of finding an effective and safe method to control crop diseases. As an endogenous phytohormone released during the stress phase, BR significantly contributes to boosting endogenous physiological activities, e.g., promoting cell enlargement and division, scavenging excessive reactive oxygen species (ROS), and regulating photosynthetic capacity to improve plant stress resistance and adaptability [39,40]. It was reported that only a low concentration of BR could effectively improve the resistance to chilling injury in corn and mango [41,42]. In tomato plants, it has been found that BR can defend the photosynthetic structure from effective oxidation damage and trigger the stability of chlorophyll in drought-induced stress through the upregulation of protective enzyme activities in leaves [43]. Furthermore, BR similarly helps in increasing photosynthesis by expanding the leaf area, increasing Rubisco activity, and enhancing nitrate reductase activities [44].

Melon has become an excellent model plant for dissecting the essential biological mechanisms involved in the regulation of important traits [45]. To date, molecular-basis studies have elucidated the functions of different candidate genes and pathways controlling disease susceptibility to stress and pathogen attacks [32]. However, information about how exogenous BR relieves the crucial damage due to downy mildew infection in cucurbit crop species is limited, and the identification of a specific BR application for controlling downy mildew resistance is still needed in melon. Herein, we tried to assess the efficacy of distinct exogenous BR treatments towards maintaining physiological parameters (photosynthetic responses and antioxidant activities) and genetic regulation for controlling downy mildew disease stress in melon.

## 2. Results

### 2.1. Analysis of Phenotypic and Cytological Changes in Leaves

**Figure 1:**
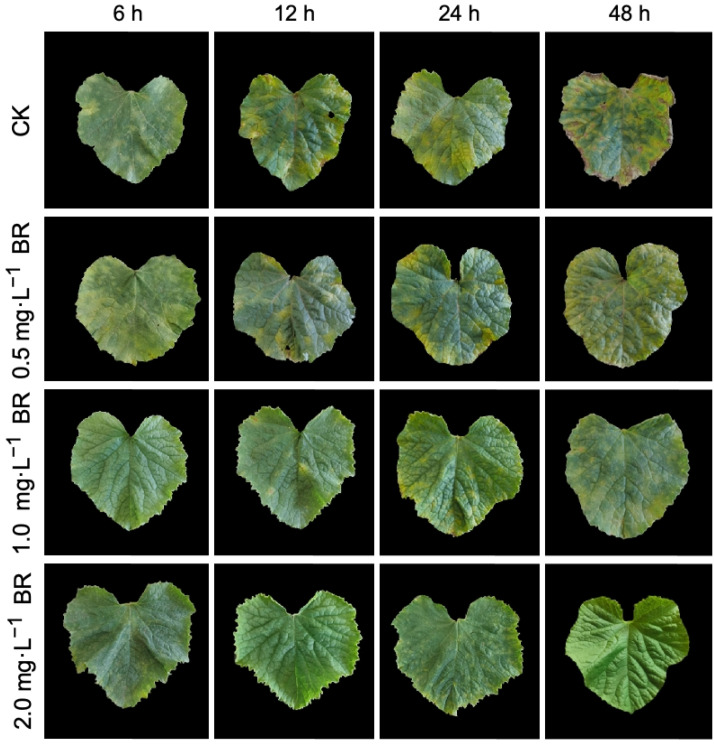
The visual observations of melon seedling leaves infected by downy mildew stress and the impact of different concentrations of exogenous BR treatment at various time intervals. CK, control (without BR treatment); BR, brassinolide; mg·L^−1^, milligrams per liter; h, hours.

To elucidate the impact of various concentrations of BR on the changes in the physiological activities of melon seedling leaves induced by downy mildew, we distinguished the disease levels followed by the above-mentioned standard. Just as the results showed (Figure 1), the propagation velocity of downy mildew on leaves was very rapid, which infected the entire leaf within 48 h in the control treatment, causing extensive yellowing and necrosis symptoms. However, the obvious effect of BR on enhancing the resistance of melon contained the spread of disease over time. The disease levels of BR-treated groups could be classified at Grade 5 or even lower. The resistance effect of 1.0 and 2.0 mg·L^−1^ BR treatments was observed to be more pronounced than that of 0.5 mg·L^−1^ treatments, and the disease spots seemed to nearly disappear under a 2.0 mg·L^−1^ BR concentration at a 48 h time interval.

**Figure 2:**
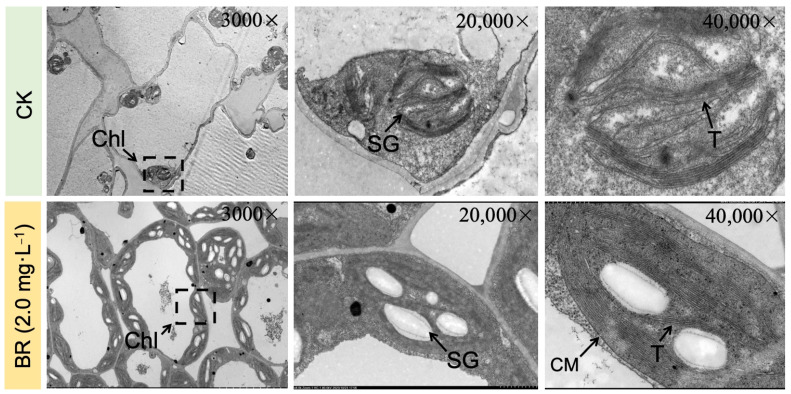
Microscopic view of internal organelle ultrastructure of DM-infected leaves of melon seedling observed under CK and exogenous BR treatment at 48 h of the time period. CK, control (without BR treatment); BR, brassinolide; Chl, chloroplast; CM, chloroplast membrane; T, thylakoid; SG, starch granule.

Transmission electron microscopy (TEM) observation was performed to examine the internal organelle ultrastructure of seedling leaves under the comparative groups (Figure 2). The chloroplast (Chl) and chromoplast (CM) membrane structures in seedling leaves of the CK (without BR application) group were shriveled and spindle-shaped; the thylakoid (T) structure was irregular and its configuration was obscure; and the starch granular (SG) content was reduced and their integrity was diminished. However, the leaf organelles in the BR (2.0 mg·L^−1^) treated group showed a proper configuration of thylakoid structure, and the content of starch granules was significantly higher compared with the CK group. Based on these results, it can be observed that downy mildew infection severely damaged the essential photosynthetic mechanism in melon seedling leaves, thus negatively affecting the metabolic pathway of photosynthesis pigments and carbohydrates, while BR treatment effectively protected the basic structure of organelles and ensured the normal progress of photosynthesis and other antioxidant activities.

### 2.2. Analysis of Enzymatic Activity of Antioxidants and Permeability of Cell Membrane

**Figure 3:**
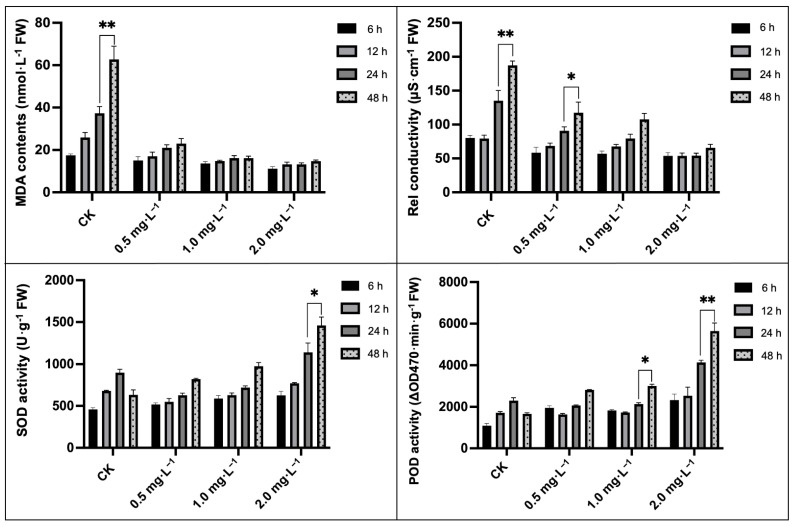
Impacts of different BR treatments on the malondialdehyde (MDA) content, relative conductivity, and SOD and POD activity of melon seedling leaves under downy mildew stress. Asterisks symbols represent the significant results at the ** *p* < 0.01 and * *p* < 0.05 levels, respectively.

It was observed that with increasing downy mildew stress, the overall MDA content was significantly enhanced by nearly eight times relative to the MDA content in leaves in the CK (without BR treatment) group compared to the BR-treated group, thereby suggesting disease-induced stress damage to the cell membrane, which was also corroborated by an improved REC value (Figure 3). It was observed that BR treatments significantly hindered these indices over the control (CK) treatment. Further, a marked rise in SOD and POD activities with downy mildew disease stress was also observed due to an interactive effect of abnormal homeostasis in cells and damaged electron transport networks, but the antioxidant capacity in CK seemed to be limited compared with the further improved enzyme activities in the 2.0 mg·L^−1^ BR treatment (Figure 3). These evidences suggested that downy mildew negatively impacted the plastid membranal structure and composition, while BR could significantly scavenge excessive MDA and activate antioxidants to maintain membrane-lipid homeostasis. Moreover, the severe loss of chlorophyll was in line with the visually detected differences in chloroplast ultrastructure shown in Figure 2.

The molar percentages of MGDG and DGDG regulated the total contents of glycerolipid in leaves and directly occupied >65% of the glycerolipid contents, while both MGDG and DGDG content decreased by 30.58% and 48.22% under downy mildew stress at 48 h compared to the other time intervals, respectively; however, it seemed that BR-treated (2.0 mg·L^−1^) seedling groups did not display an obvious increase in glycerolipid contents or the MGDG-to-DGDG ratio (Table 1). Thus, we suspected that the enhanced resistance triggered by BR treatment was mainly due to its antioxygenic property and its inspiration for antioxidant activities.

### 2.3. Analysis of Chlorophyll and Carotenoids Contents

**Figure 4:**
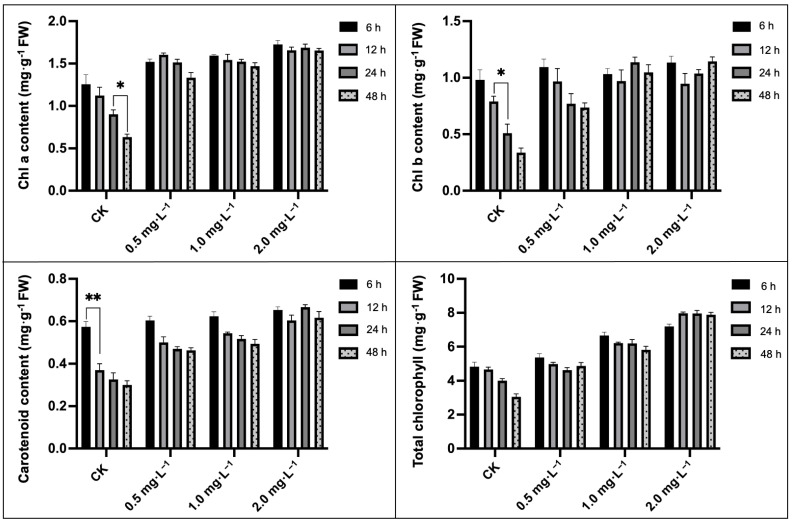
Impacts of different BR treatments on photosynthetic pigments of melon seedling leaves under downy mildew stress. Asterisks symbols represent the significant results at the ** *p* < 0.01 and * *p* < 0.05 levels, respectively.

The total chlorophyll (Chl), chlorophyll a (Chl a), chlorophyll b (Chl b), and carotenoids contents decreased significantly with the duration of downy mildew infection (Figure 4), which was in line with the ever-increasing super-oxidized photosynthesis apparatus. It was observed that the carotenoids, total chlorophyll, chlorophyll a, and chlorophyll b contents decreased by 36.92%, 50.40%, 65.31%, and 47.36% at 48 h compared with 6 h and other time intervals, respectively. The exogenous application of 2.0 mg·L^−1^ BR significantly boosted photosynthetic pigmentation in leaves more than that of CK treatment; however, there were no evident alteration of the photosynthetic pigmentation contents within different concentrations of treatments, but every physiological index was maintained at a normal level, except for a notable total chlorophyll content, which increased nearly three times (264.52%).

### 2.4. Analysis of Soluble Sugar, Starch Content, and Rubisco Activity

**Figure 5:**
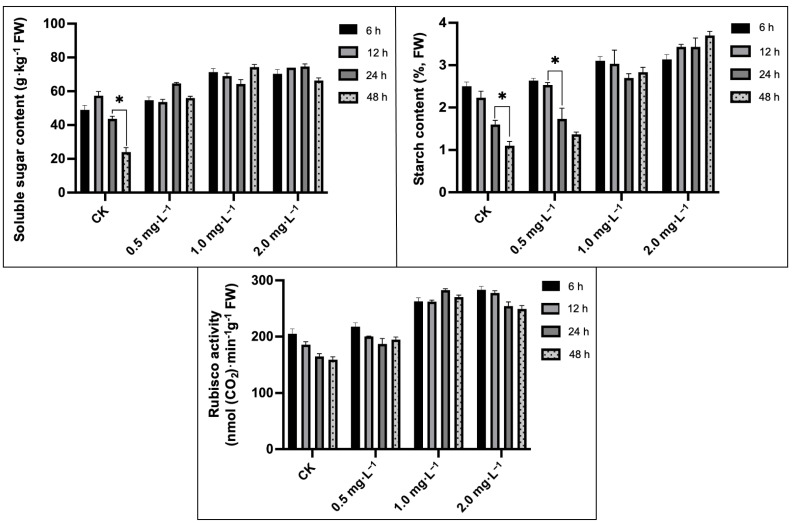
Impacts of different BR treatments on soluble sugar, starch content, and Rubisco activity of melon seedling leaves under downy mildew stress. Asterisks symbols represent the significant results at the * *p* < 0.05 level.

The major differences were observed for the soluble sugar and starch content in melon seedling leaves cultured under downy-mildew-induced stress (Figure 5). The downy mildew infection caused a 42.80% and 53.06% decrease in the starch content and soluble sugar in chlorotic leaves at 48 h compared to 6 h in the CK (control) group. In contrast, exogenous BR treatment relatively maintained the soluble sugar content, whereas no major differences were observed within different concentrations of BR treatments. Notably, starch contents showed an increasing trend between 24 h and 48 h, and the highest starch synthesis in the leaf was noticed in the BR treatment (2.0 mg·L^−1^) at 48 h. Further, it was noted that the 2.0 mg L^−1^ BR concentration significantly improved the Rubisco activity (increased by 62.89%) at 48 h compared with the CK group treatments.

### 2.5. Analysis of Variations in Chlorophyll Fluorescence Parameters

The obtained results depicted that chlorophyll fluorescence-related indices in melon seedling leaves altered severely with the increase in downy mildew stress (Table 2). It was observed that the levels of F0, Fv/Fm, NPQ, and Y (NPQ) decreased by 15.06%, 28.75%, 39.58%, and 31.11% after 48 h of disease stress, but no marked changes in the qP value were found compared with the 6 h within CK groups, respectively. While the exogenous effects of BR treatments showed an obvious concentration-dependent effect on these parameters, it was observed that 2.0 mg·L^−1^ BR treatments effectively maintained the maximum levels of F0 and Fv/Fm (42.23% and 36.67%), and nearly three times higher levels of NPQ (375.86%) and Y (NPQ) (287.10%) were also observed when compared with the treated groups at 48 h and other groups. However, the protective property of BR is not comprehensive; the slight fluctuation in Y (II) and decreased qP values indicated that BR could also inhibit some fluorescence parameters.

### 2.6. Analysis of Relative Genes Expression Trends

**Figure 6:**
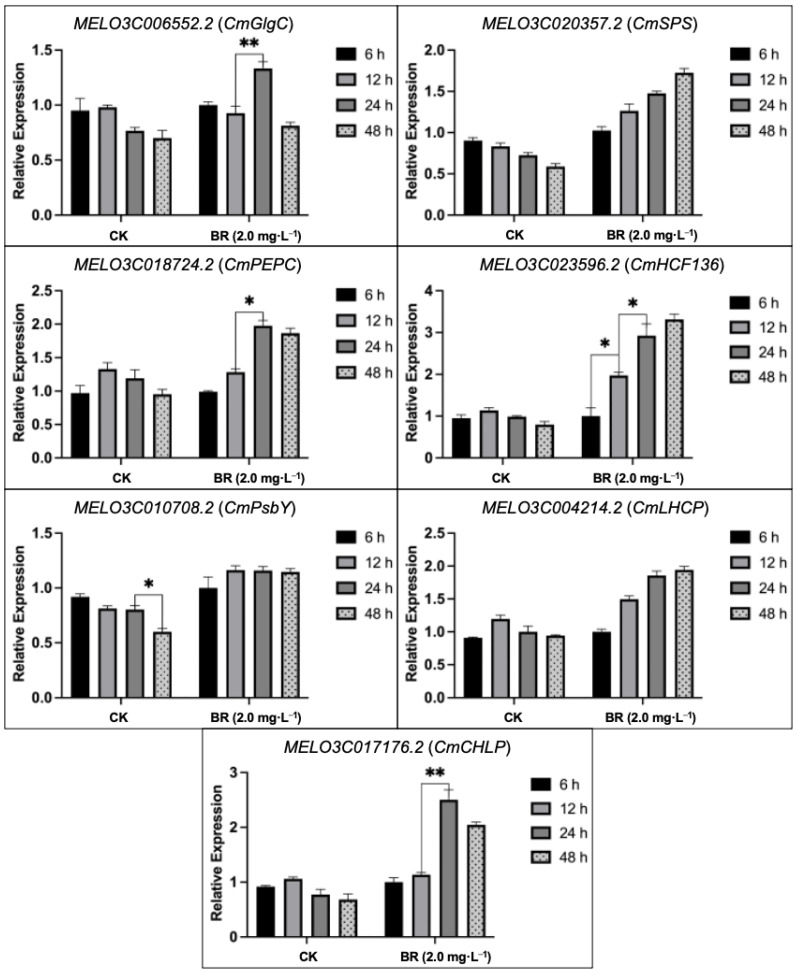
Relative expression trends of predicted genes regulating photosynthesis and antioxidant activities within the CK- and BR-treated groups under downy mildew stress. CK (control, without BR treatment); BR, brassinolide; h, hours. Asterisks symbols represent the significant results at the ** *p* < 0.01 and * *p* < 0.05 levels, respectively.

We examined the expression profiles of putative genes using qRT-PCR analysis in sampled seedling leaves of CK- and BR-treated groups at different time intervals (6 h, 12 h, 24 h, and 48 h) (Figure 6). The predicted melon genes related to photosynthetic pigment biosynthesis, PSII reaction center subunits, and carbohydrate metabolism showed a downward trend to some extent, particularly in terms of the PSII reaction center and chlorophyll biosynthesis, which decreased by 35.79% and 24.92%, respectively. However, the expression trend of the *CmHCF136* gene (which had the maximum upregulation value) was up to three times higher at 48 h compared with CK (control, without BR treatment), *CmGlgC*, *CmSPS*, and *CmCHLP*, which were significantly upregulated in the BR-treated groups. In addition, the relative stable expression levels of other genes (*CmPEPC*, *CmLHCP*, and *CmPSBY*) demonstrated that BR application has protective characteristics in enhancing photosynthesis and carbohydrate metabolism on a molecular level.

## 3. Discussion

Downy mildew is one of the most destructive diseases, disturbing the biomass and yield of the melon crop. The pathogen of downy mildew can attack leaves, and this disease is supposed to be controlled by applying fungicides that directly affect pathogen intensity, develop resistance, and effectively lead to the normal growth and development of crop plants [1]. Brassinolide serves as a new type of plant growth regulator that has been widely used to improve plant stress resistance due to its high efficiency, broad spectrum, and non-toxicity [12,46].

In the current experimental study, we tried to check the enhancing impacts of BR treatments on photosynthesis, as well as the antioxidant activity of melon seedling cultures under downy-mildew-induced infection. It was significantly observed that the melon seedlings showed leaf-yellowing symptoms on leaves with the extended time period of DM stress (Figure 1), and similarly, the internal photosynthesis-related organelle structure was severely damaged (Figure 2). However, we observed that exogenous 2.0 mg·L^−1^ BR treatment effectively enhanced the biochemical adaptations of the melon plant towards the pathogen infection of DM disease. Based on the antioxygenic property and the elevated activities of the antioxidant enzymes catalyzed by BR, a greater antioxidation capacity has been endowed to melon for resisting downy mildew infection. Further, it was seen that 2.0 mg·L^−1^ BR triggered a significant impact on the inhibition of the development of DM disease compared with CK (Figure 1) and protected the photosynthetic apparatus and carbon metabolic pathway, which were measured by the membrane-lipid components, MDA and REC values, pigment concentrations (Figure 3 and Figure 4), chlorophyll fluorescence parameters (Table 2), and the upregulated expression trend of genes related to carbon fixation (Figure 6). A similar pattern of BR response with an elevation in photosynthesis and plant biomass was observed in maize plants grown under chilling stress [47]. Ali et al. [48] also reported that 24-epiBL enhanced the level of the antioxidant system under stress and normal conditions, while the effect was more pronounced under stress situations. Thus, we suppose that the influence of BR treatment on improving the protective machinery of melon deserves further promotion in the cultivation process.

Regarding the physiological activities in leaves, Fazeli et al. [49] observed that downy-mildew-induced infection led to numerous ROS accumulation in cells and caused severe super-oxidized damage to the membrane in plants. However, excessive ROS could be scavenged by the antioxidant protective enzymes [50]. Tommasino et al. [25] reported that SOD and POD play a main role as protective enzymes in the resistance mechanism towards catalyzing the conversion of O^2−^ to H_2_O; however, the oxidant scavengers and MDA content (which are substantially released when the cell membrane is damaged) could be used jointly as essential indicators to reflect the degree of membrane-lipid peroxidation that is caused by the ROS [48]. In the present study, the internal structure of organelles and membranes has been seriously attacked by downy mildew infection (Figure 2) and the obviously increased MDA content (Figure 3). It seemed that the upregulation of antioxidant activities during the initial period was also in line with this trend. However, with the gradual development of disease stress, the antioxidant activity reached its minimum limit and remained at a stable decreased level. While the application of BR treatments effectively enhanced the SOD and POD activities and markedly decreased the MDA content (Figure 3), we also found a significant effect of BR on SOD activity, which also depicted an apparent effect greater than that on POD, and the results are in line with Chen et al. [51], who reported the significant enhancing effect of BR application on physiological parameter regulation in drought tolerance. The previous study reported that BR treatment could regulate and upsurge the endogenous activities of antioxidant enzymes in plants grown under adverse stress phases. These activities take place through the genetic modulation of protein configuration or the functional expression of enzyme genes in soybean plants [52]. Aghdam et al. [53] found that brassinosteroids treatment had a significant enhancing impact on PAL activity and flavonoid contents in tomatoes during post-harvest cold storage. It was also reported that radish seed treatment with 24-epibrassinolide (0.001 μmol L^−1^) could improve the antioxidant activities, as well as restrain the free radical capacity in seed sprouts under copper ion (Cu^++^) stress [54]. Li et al. [55] reported that brassinolide treatment improved the POD activity, SOD activity, and ABTS levels in radish sprouts preserved at a low temperature. Cao et al. [56] revealed that BR elevated SOD and POD activities by enhancing the expression of the *DET2* gene. Thus, we speculate that the elevated resistance in downy-mildew-susceptible plants treated by BR may be attributed to oxidative stress defense mechanisms.

Chlorophyll is a pigment that synthesizes green color in plants and helps to produce food through the photosynthesis process and the accumulation of internal pigment-protein structures. While the chloroplast is a candidate source of ROS in plant cells, severe degradation of chlorophyll takes place as ROS levels increase during the stress phase [57]. The biosynthesis pathway of chlorophyll relies on various proteins, and any genetic variation can have a negative influence on chlorophyll synthesis pathways. Tanaka et al. [58] reported that the tetrapyrrole biosynthesis pathway (TBP) is a major activity during chlorophyll production, and the biosynthesis of 5-aminolevulinic acid (ALA) and magnesium chelase (MgCh) are essential points regulating the chlorophyll synthesis activity. MgCh is a multifaceted subunit composed of *CHLH*, *CHLP*, and *CHLD*, and it mainly involves the initial stage of the branch reaction of chlorophyll biosynthesis [58]. Our current study results depicted that the exogenous BR treatment effectively increased the synthesis of chlorophylls a and b (Figure 4), and the expressions of *CmCHLP* and *CmLHC*, whose products are connected to chlorophyll a and b, were all downregulated by downy mildew (Figure 6), which may have explained the diminished chlorophyll content. Carotenoids are organic pigments that are distributed around chlorophyll and help to absorb solar energy as well as transfer it to chlorophyll. Meanwhile, carotenoids also have the function of protecting the photosynthetic organs from damage and maintaining chloroplast integrity and enhancing the membrane antioxidant activity [59,60,61]. In our study, carotenoids synthesis increased at the beginning of stress, contrary to the continuous decrease in chlorophyll content (Figure 4), which is similar to the former stated result of Tattini et al. [62]. Further, we observed that the exogenous BR plays a role in triggering chlorophyll biosynthesis by upregulating the expression of *CmCHLP* and *CmLHC*, as well as relieving the downward trend of Chl a and Chl b (Figure 6), and thereby protecting photosynthetic chloroplasts against the downy-mildew-induced destruction of pigments.

The membrane is an important intracellular barrier between plants and external stimuli that relies on its distinct configuration, permeability, and other protective features [11]. MGDG and DGDG are the main membrane-lipid compositions that significantly regulate the chloroplast structure and photosynthetic properties, while the lipid content would remodel to enhance membranal stability and integrity when exposed to stress conditions [16,20,21]. In our study results, downy mildew induced significant lipid remodeling in the membrane system, which was in line with the decreased photosynthetic pigment content. This indicated that extreme ROS caused by disease stress adversely affected the structure of the plastid membrane and its arrangement, similarly disrupting the subsequent metabolic process. In addition, the application of BR did not cause an obvious alteration in the molar percentages of galactolipids compared with the CK group, and the content of each component was maintained at a relatively stable level (Table 1). Therefore, the protective mechanism of BR may also profit from its ROS scavenging capacity.

Photosynthesis is the most basic biological activity in cellular organisms and also a complicated metabolic process that allows plants to convert light energy into chemical energy in their normal growth and development phases, but it is affected by climate change stress [63]. Ribulose bisphosphate carboxylase/oxygenase (Rubisco), a protein that plays a vital role in photosynthesis, takes responsibility for the first chemical reaction of the Calvin cycle. The greatest biological significance of Rubisco lies in its function to catalyze the conversion of inorganic carbon to biomass, which completes the carbon cycle from the outside to the inside of plants. In addition, SPS plays a vital role as a key enzyme in the final sucrose synthesis and catalyzes fructose-6-phosphate and uridine diphosphate glucose (UDPG) for synthesizing sucrose-6-phosphate, which delivers a substrate for sucrose phosphatase to catalyze sucrose synthesis [64]. Meanwhile, phosphoenolpyruvate carboxylase (PEPC) is a controlled enzyme situated at the core of plant C-metabolism that enhances the irreversible β-carboxylation of PEP to form a series of enzymatic reaction substrates [65]. Our research exhibited that the application of exogenous BR can effectively improve Rubisco activity (Figure 5), as well as the expression of predicted genes (*CmGlgC*, *CmPEPC*, and *CmSPS*) **(**Figure 6) which regulate carbon fixation and assimilation, ultimately upsurging the soluble sugar content.

Soluble sugar is also an important stress regulator, and signaling molecules are involved in the plant defense mechanism against adversity via carbon storage and free radical scavenging [66]. The increased soluble sugar content in BR treatment efficiently relieved the toxicity of downy mildew (Figure 5). Altogether, exogenous BR obviously improved the resistance mechanism towards downy mildew stress in melon and alleviated the disease effect on the carbon metabolism procedure. The PSII reaction center may be the primary target for injuries from various stress conditions. Zhang et al. [33] suggested that photosynthesis (PSII) in pepper plants constantly bears photo-inhibition during low-temperature stress. Furthermore, our previous study revealed that thylakoid membranes were extremely super-oxidized under salt stress [32], thus significantly inhibiting the light harvesting and absorption processes. In the present study, downy mildew induced lipid remodeling in thylakoid, which contains most of the photosynthetic pigment, causing chlorotic spots in leaves and further affecting photosynthetic efficiency (Figure 2). Li et al. [67] stated that Psb protein-induced mechanisms can alleviate the PSII structure under salinity stress. In addition, *HCF136* encodes a hydrophilic protein localized in stromal thylakoids and plays an essential role in assembling the PSII reaction center in Arabidopsis [34]. Herein, qRT-PCR results revealed that downy mildew caused a downregulated expression in genes (*CmHCF136* and *CmPsbY*), while BR treatment effectively alleviated the downward trend of these genes to protect the stability of the PSII reaction center (Figure 6).

The chlorophyll fluorescence parameter is an important measurement to determine specific photosynthetic indices under stress conditions [68,69,70,71]. The F0 value is the least fluorescence that denotes the basis of the photosystem II (PSII) reaction system [72,73]. Our current findings depicted that the F0 values in melon seedling leaves decreased at 48 h post-infection, while the samples treated with BR showed higher F0 values compared to the CK group (Table 2), indicating that downy mildew might directly damage the PSII system during the disease period and that activation of the PSII reaction center towards light was diminished in melon leaves. Li et al. [67] found that 24-epibrassinolide application could relieve the photo-inhibition by upsurging Fv/Fm florescence in pepper seedlings under low-temperature conditions. BR treatment could relieve the crucial damage to some extent in crop plants. The Fv/Fm value could serve as an indicator to reflect the maintained PSII function and photosynthesis, while the decreased Fv/Fm parameter denotes the reduction in photosynthetic pigment in plants affected by stress induction. Meanwhile, the maximum NPQ value indicates a stronger photoprotective capacity in crop plants [74,75,76]. Kaya et al. [74] reported that exogenous foliar sprays of 24-epibrassinolide (1 μmol/L) clearly enhanced the Fv/Fm parameter under water deficit stress but did not have an obvious influence on the Fv/Fm parameter under regulated water management. Herein, the Fv/Fm and NPQ parameters in melon leaves were improved by overall BR treatments against downy mildew stress compared with the CK group (Table 2). However, the enhancement efficiency of BR seemed limited to getting these parameters back to normal levels, and it could not further improve these parameters. Therefore, we propose that the photoprotective mechanisms of BR are based on its anti-oxygenation, so that it is more suitable for acting as a healing agent when matched with other bio-medicines. In contrast to the above-mentioned parameters, our study indicated that BR treatment did not increase the significant levels of qP and Y(II) in leaves, and even exacerbated the downward trend, which was similar to the result of Xue et al. [75]. Y(II) indicates the definite light energy change efficacy of PSII, while qP values expose the photosynthetic activity (PSII reaction system) in plants through chlorophyll pigmentation [77]. Thus, we speculated that exogenous BR might not directly participate in the energy conversion, while it might resist photoinhibition by distributing the energy in the PSII system by assisting in enhancing the aptitude for photoprotection.

## 4. Materials and Methods

### 4.1. Plant Materials and Downy-Mildew-Induced Stress

The melon variety (Longqing, susceptible to downy mildew disease) was used as the tested plant material, and the plant hormone (brassinolide, BR), used as an exogenous treatment, was purchased from Aladin Laboratory Technologies Inc. (Shanghai, China). The melon seedlings were cultivated by adopting a completely randomized design (CRD) in the controlled environment of a greenhouse located at the Daqing Branch of the Horticulture Laboratory of Heilongjiang Academy of Agricultural Sciences (HAAS), Heilongjiang Province, China.

To ensure the seed germination and proper cultivation of melon seedlings, the seeds were first disinfected by sterilizing in a 0.5% solution of sodium hypochlorite (NaOCl) for a maximum of 5 min and washed with distilled water (ddH_2_O) to remove the residues. Then, all the seeds were placed into a wet germination tray at 25 °C/22 °C under a photoperiod cycle of 16 h/light and 8 h/dark. The melon seedlings were grown in a greenhouse at the required temperature range of 23 °C–28 °C and humidity of 50–80%. Twenty-day-old (about three leaf stage) seedlings were artificially infected with fungal spores of downy mildew disease and divided into two respective groups: the first group was simply sprayed with distilled water (ddH_2_O) to retain the moisture and marked as control (CK, without BR treatment); the second group was exogenously sprayed with different treatments of slightly modified concentrations of BR solution (0.5, 1.0, and 2.0 mg·L^−1^) [55], to check the effectiveness of BR treatment for maintaining the growth and physiological indices of the melon plant against downy mildew disease stress. Then, the developed seedlings were inspected regularly based on the development of DM symptoms, and leaves were collected at specific time periods of 6 h, 12 h, 24 h, and 48 h from the CK- and BR-treated groups. The collected samples were cooled using liquid nitrogen and quickly preserved for the subsequent determination of physiological activities and molecular experimentation. A total of three replications were used for each treatment, and parameters were recorded correspondingly.

### 4.2. Observation of Disease Symptoms and Transmission Electron Microscopy

The disease-symptom levels in downy-mildew-infected leaves of melon seedlings were observed according to the distinguished grades, as followed by Gao et al. [78]: Grade 0: leaves without visual disease spots; Grade 1: about <5% visual disease spots on the whole leaf. Grade 3: about 6–10% visual disease spots on the whole leaf; Grade 5: about 11–25% visual disease spots on the whole leaf; Grade 7: about 26–50% visual disease spots on the whole leaf; Grade 9: about >50% visual disease spots on the whole leaf, respectively.

For the transmission electron microscopy (TEM) analysis, the samples were collected from the normal developing and diseased parts of the pathogen-infected leaves of the control and 2.0 mg·L^−1^ BR treatment at 48 h. The cytological observations of the internal organelle structure were observed under normal room light using a high-quality screen camera of the transmission electron microscope (JEOL, JEM-1200EX, Tokyo, Japan) by following the detailed method of Liu et al. [32].

### 4.3. Evaluation of Antioxidant Enzymatic Activity and Cell Membrane Permeability

The fresh leaves were sampled from the CK- and BR-treated groups at different time periods (0 h, 6 h, 12 h, 24 h, and 48 h) under downy-mildew-induced stress, and the antioxidant enzymatic activity was evaluated. The lipid peroxidation was estimated based on the malondialdehyde (MDA) content within leaf homogenates treated with 2-thiobarbituric acid (TBA), as reported earlier by Liu et al. [79].

The relative electrical conductivity (REC) value was calculated in percentage by following the earlier reported method [79]: REC (%) = EC1/EC2 × 100%, whereby EC1 and EC2 represented both electrical conductivities (first and final), respectively.

The superoxide dismutase (SOD) was isolated, and the final SOD activity was assessed using the spectrophotometer (DU520 UV/VIS, Thermo Fisher Scientific, Delaware, DE, USA) following colorimetric evaluation at a 560 nm absorption rate for the extract. The SOD measurement unit was per gram (g) of fresh weight (FW) per minute [80].

The peroxidase (POD) activity was assessed using a spectrophotometer (DU520 UV/VIS, Thermo Fisher Scientific, Delaware, DE, USA) following the alterations in optical density at 240 nm at 30 s intervals in the biochemical reaction. The POD measurement unit was per gram (g) of fresh weight (FW) per minute [80].

Lipid isolation from melon seedlings leaves was conducted using the modified protocol of Narayanan et al. [81].

### 4.4. Estimation of Total Soluble Sugar, Starch Content and Rubisco Activity

A total of 0.1 g of fresh leaf samples was weighed and ground in an ice bath, and Rubisco activity was determined by using the Rubisco Kit (Ge Ruisi, Suzhou, China) [82]. Then, a total of 0.5 g of leaf samples was isolated in hot alcohol (80%), and an alcohol-free liquid isolate was used for the estimation of total sugar and starch contents, as reported earlier [83].

### 4.5. Determination of Photosynthetic Pigment Content

The photosynthetic pigments (total chlorophyll, chlorophyll a, chlorophyll b, and carotenoids) were determined using the 80% ethanol–acetone extraction protocol. The leaf tissue samples (0.3 g fresh weight (FW)) were collected from comparative groups of the same size using a puncher and soaked in an equal ratio (1:1) of ethanol–acetone solution for about 24 h. The spectrophotometer (DU520 UV/VIS, Thermo Fisher Scientific, Delaware, DE, USA) was used to measure the absorbance of the extract, and measurements were recorded as mg·g^−1^ fresh weight (FW) [84,85].

### 4.6. Determination of Photosynthetic Fluorescence Parameters

The significant changes in chlorophyll fluorescence parameters are not easily observed by the naked eye. To evaluate the impacts of downy-mildew-induced stress on the chlorophyll fluorescence parameters of melon seedling leaves, we used an automated LI-COR LI-6400XT portable photosynthesis system (LI-COR Biosciences, Lincoln, NE, USA) to measure the minimal fluorescence (F0), the maximum photochemical efficiency of PSII (Fv/Fm), the quantum efficiency of PSII photochemistry (Y(II)), the non-photochemical quenching coefficient (NPQ), and the quantum yields of regulated energy dissipation Y(NPQ) of the control and BR-treated groups, respectively [86].

### 4.7. Validation of Expression Trends of Putative Genes

Firstly, total RNA was isolated from collected leaf samples at 0 h, 6 h, 12 h, 24 h, and 48 h using the Simply P Total RNA Extraction Kit BSC60 (Bioer Technology, Hangzhou, China), and three independent biological replicates for each measurement were included, respectively. The first strand complementary DNA (cDNA) was synthesized from 2 μg of RNA template using GoScript^TM^ Reverse Transcriptase (Madison, WI, USA), and the Novostar-SYBR Supermix (Novoprotein, Suzhou, China) was used to perform qRT-PCR analysis on the Bio-Rad CFX96^TM^ Connect Optics Module (Hercules, CA, USA). The 2^−ΔΔ*CT*^ formula was used to estimate the fold changes in the relative gene expression trend [87] through qRT-PCR analysis between samples of the control and BR-treated groups, and *Actin* was used as a housekeeping gene. The associated putative genes used in this experiment were predicted and obtained based on the few earlier reported genes and their annotations [34,64,65,84,88,89,90] as shown in Table S1. Then, the functional annotation of each gene was searched and predicted across the melon genome (DHL92, v3.6.1) dataset available at the website of the Cucurbit Genomics Database (CuGenDB, (http://cucurbitgenomics.org/search, accessed on 10 June 2023)). Detailed information on the full-length gene sequences and exported primers can be seen in Table S2, respectively.

### 4.8. Statistical Data Analysis

All the physiological parameters were measured accurately on a regular basis, and data analysis was performed using statistics software (IBM SPSS, version 26.0). The significant differences among the obtained results were observed by performing the Duncan’s test at two levels of significance (** *p* < 0.01 and * *p* < 0.05 levels), respectively.

## 5. Conclusions

Herein, we effectively demonstrated the enhancing effects of exogenous BR on improving the downy mildew resistance mechanism for photosynthesis and carbon metabolism in the melon plant. The obtained results clearly showed that downy mildew induced a severe imbalance of physiological function and caused a significant reduction in photosynthesis, membranal stability, and enzyme activity. The PSII reaction system seems to be the primary target to accept stress, as revealed by the alterations of membrane lipids and the obviously downregulated expressions in *CmHCF136* and *CmPsbY*. The inhibition effects of *CmCHLP* and *CmLHC* induced by downy mildew directly caused the degradation of chlorophyll and resulted in chlorotic spots in leaves. However, the exogenous application of BR treatment at 2.0 mg·L^−1^ significantly reduced the disease index and enhanced antioxidant activities to eliminate excessive ROS and MDA contents, thereby contributing to stabilizing the intrinsic properties of the photosynthetic phenomenon and chlorophyll fluorescence parameters. The upregulated expressions of *CmGlgC*, *CmPEPC*, and *CmSPS* increased the Rubisco activity and enhanced the resistance of melon plant leaves to disease attack. In summary, we believe that our research findings exposed the effective BR concentration (2.0 mg·L^−1^) in controlling the physiological indices and genetic regulation of plants under downy mildew disease stress, but in-depth studies at the transcriptional and translational levels are still needed for further validation on a large scale.

## Figures and Tables

**Table 1:** Molar percentage of DGDG and MGDG in CK- and BR-treated groups of melon seedling leaves at different time periods. CK, (control, without BR treatment); BR, brassinolide; h, hours; DGDG, digalactosyldiacylglycerol; MGDG, monogalactosediacylglycerol. Statistical letters represent the significant differences among the observed values, respectively.

Molar (%)	CK				BR (2.0 mg·L^−1^)			
	6 h	12 h	24 h	48 h	6 h	12 h	24 h	48 h
DGDG (%)	26.73 ± 0.89 ^a^	22.80 ± 1.04 ^a^	17.65 ± 2.04 ^a^	13.84 ± 3.02 ^ab^	25.40 ± 1.12 ^a^	26.15 ± 0.75 ^a^	25.31 ± 0.80 ^a^	26.03 ± 0.85 ^a^
MGDG (%)	38.68 ± 1.67 ^a^	34.21 ± 2.23 ^b^	31.07 ± 0.77 ^a^	26.85 ± 2.80 ^b^	40.69 ± 1.03 ^a^	38.76 ± 1.18 ^a^	41.28 ± 1.57 ^a^	40.03 ± 0.41 ^a^

**Table 2:** Exogenous impacts of various BR treatments on chlorophyll fluorescence parameters of melon seedling grown under downy mildew stress. CK, (control, without BR treatment); BR, brassinolide; h, hours. Statistical letters represent the significant differences among the observed values, respectively.

Treatments	Duration	NPQ	Y (NPQ)	qP	F0	Fv/Fm	Y (II)
CK	6 h	0.48 ± 0.09 ^a^	0.45 ± 0.09 ^a^	0.50 ± 0.04 ^a^	75.33 ± 0.07 ^a^	0.80 ± 0.09 ^a^	0.36 ± 0.04 ^a^
	12 h	0.59 ± 0.04 ^a^	0.53 ± 0.04 ^a^	0.48 ± 0.07 ^a^	78.26 ± 0.12 ^a^	0.76 ± 0.11 ^a^	0.38 ± 0.07 ^a^
	24 h	0.32 ± 0.12 ^a^	0.34 ± 0.12 ^a^	0.40 ± 0.06 ^a^	70.87 ± 0.09 ^ab^	0.65 ± 0.08 ^a^	0.41 ± 0.06 ^a^
	48 h	0.29 ± 0.06 ^a^	0.31 ± 0.06 ^a^	0.49 ± 0.03 ^a^	63.68 ± 0.10 ^a^	0.57 ± 0.10 ^a^	0.38 ± 0.12 ^b^
0.5 mg·L^−1^ BR	6 h	0.53 ± 0.10 ^a^	0.50 ± 0.10 ^a^	0.40 ± 0.10 ^a^	80.69 ± 0.02 ^a^	0.78 ± 0.05 ^a^	0.40 ± 0.10 ^a^
	12 h	0.79 ± 0.04 ^a^	0.74 ± 0.04 ^a^	0.48 ± 0.07 ^a^	79.44 ± 0.13 ^a^	0.83 ± 0.16 ^ab^	0.37 ± 0.07 ^a^
	24 h	0.83 ± 0.06 ^a^	0.80 ± 0.06 ^a^	0.50 ± 0.15 ^b^	82.37 ± 0.28 ^c^	0.85 ± 0.10 ^a^	0.45 ± 0.15 ^b^
	6 h	1.14 ± 0.02 ^a^	1.02 ± 0.16 ^c^	0.46 ± 0.01 ^a^	85.20 ± 0.07 ^a^	0.86 ± 0.07 ^a^	0.38 ± 0.01 ^a^
1.0 mg·L^−1^ BR	6 h	0.47 ± 0.07 ^a^	0.50 ± 0.07 ^a^	0.48 ± 0.04 ^a^	76.70 ± 0.15 ^a^	0.83 ± 0.15 ^a^	0.43 ± 0.07 ^a^
	12 h	0.66 ± 0.10 ^c^	0.59 ± 0.10 ^c^	0.38 ± 0.10 ^b^	80.50 ± 0.07 ^b^	0.88 ± 0.07 ^b^	0.40 ± 0.13 ^c^
	24 h	0.89 ± 0.07 ^b^	0.85 ± 0.07 ^b^	0.36 ± 0.12 ^b^	90.45 ± 0.18 ^b^	0.92 ± 0.08 ^a^	0.36 ± 0.11 ^a^
	6 h	1.25 ± 0.08 ^b^	1.14 ± 0.08 ^b^	0.41 ± 0.03 ^a^	91.66 ± 0.03 ^a^	0.90 ± 0.03 ^a^	0.42 ± 0.08 ^a^
2.0 mg·L^−1^ BR	6 h	0.52 ± 0.10 ^b^	0.57 ± 0.10 ^b^	0.33 ± 0.09 ^a^	79.65 ± 0.13 ^a^	0.91 ± 0.13 ^a^	0.38 ± 0.07 ^a^
	12 h	0.94 ± 0.27 ^c^	0.90 ± 0.27 ^c^	0.38 ± 0.05 ^a^	88.41 ± 0.57 ^c^	0.88 ± 0.04 ^a^	0.33 ± 0.10 ^b^
	24 h	0.87 ± 0.14 ^a^	0.91 ± 0.14 ^a^	0.40 ± 0.05 ^a^	85.40 ± 0.41 ^c^	0.90 ± 0.11 ^a^	0.42 ± 0.11 ^b^
	6 h	1.38 ± 0.15 ^b^	1.21 ± 0.15 ^b^	0.42 ± 0.02 ^a^	90.57 ± 0.13 ^a^	0.95 ± 0.08 ^ab^	0.40 ± 0.02 ^a^

## Data Availability

The original contributions presented in the study are included in the article and Supplementary Materials.

## Supplementary Materials

The following supporting information can be downloaded at https://www.mdpi.com/article/10.3390/plants13060779/s1, Table S1: The information of previously predicted genes used for verification in melon using qRT-PCR analysis in this study; Table S2: The information of gene ID, gene sequence, and exported primer sequences, respectively.
